# Small bowel diverticula in elderly patients: a case report and review article

**DOI:** 10.1186/s12893-022-01541-y

**Published:** 2022-03-18

**Authors:** Marah Mansour, Yazan Abboud, Racha Bilal, Nour Seilin, Tamim Alsuliman, Fawaz K. Mohamed

**Affiliations:** 1 Faculty of Medicine, Tartous University, Tartous, Syrian Arab Republic; 2 Karsh Division of Gastroenterology and Hepatology, Cedars-Sinai Medical Center, Los Angeles, CA USA; 3 Faculty of Medicine, Damascus University, Damascus, Syrian Arab Republic; 4 Department of Internal Medicine, Damascus Hospital, Damascus, Syrian Arab Republic; 5 Hematology and Cell Therapy Department, Saint-Antoine Hospital, AP-HP, Sorbonne University, Paris, France; 6 Department of General Surgery, Al-Basel Hospital, Tartous, Syrian Arab Republic

**Keywords:** Small bowel diverticula, Complicated small intestinal diverticulosis, Jejunal diverticulum perforation, Diverticulectomy, Case report, Review article

## Abstract

**Background:**

Small intestine diverticula are rare findings that were mostly reported in the elderly population as asymptomatic findings. However, they can also present with a wide range of symptoms (bloating, early satiety, chronic abdominal discomfort, and diarrhea/steatorrhea) or complications (gastrointestinal bleeding, small bowel obstruction, acute diverticulitis, or perforation) which in turn warrant medical treatment or urgent surgical intervention.

**Case presentation:**

This is a case report of an 84-year-old female who presented with an acute surgical abdomen. An exploratory laparotomy revealed complicated small bowel diverticula with a jejunal diverticulum perforation, for which a diverticulectomy was performed.

**Conclusions:**

Throughout this paper, we are aiming to outweigh the consideration of the possibility of complicated small bowel diverticula as a differential in the evaluation of any acute abdomen, especially in the elderly, which warrants emergency surgical management.

## Background

Excluding Meckel’s diverticulum, small bowel diverticula are rare findings that have been reported anywhere in the small bowel, with the duodenum being the most common site. This latter is followed to a much lesser extent by the jejunum or ileum, and lastly with the three locations simultaneously combined [1]. Its prevalence rises with age, peaking in the 50–70s [2]. While diverticula can be classified as congenital or acquired, non-Meckel diverticula are mostly acquired pseudodiverticula, composed of mucosa, submucosa, and serosa only. Their exact etiology has not been definitively identified. However, intestinal dysmotility and the structural weakness of penetration areas of the vasa recta blood vessels and nerves have been thought to play a role [3]. Most small bowel diverticula patients are asymptomatic. Nevertheless, some may present with chronic symptoms such as bloating, early satiety, chronic abdominal discomfort, diarrhea/steatorrhea due to bacterial overgrowth, or with complications such as gastrointestinal bleeding, small bowel obstruction, obstructive jaundice/recurrent pancreatitis, acute diverticulitis, or perforation [3]. Small bowel diverticula can be visualized on contrast imaging of the gastrointestinal tract, Computed Tomography (CT) scans, and Magnetic Resonance Imaging (MRI), or intraoperatively. Asymptomatic patients do not need treatment. However, the management of symptomatic cases depends on the clinical presentation (e.g., antibiotic therapy in cases of diarrhea and malabsorption caused by bacterial overgrowth, Endoscopic Retrograde Cholangiopancreatography (ERCP) in choledocholithiasis, and surgery in the acute abdomen presentation) [3–5]. Throughout this paper, in the light of a literature review, we describe a case of an 84-year-old female with complicated small bowel diverticula.

## Case presentation

**Fig. 1:**
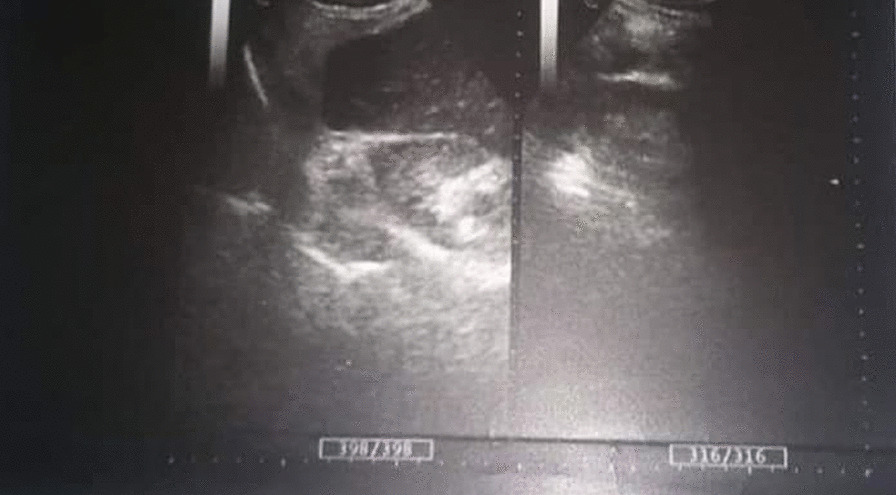
Abdominal Ultrasound demonstrating a normal liver and a resected gallbladder

**Fig. 2:**
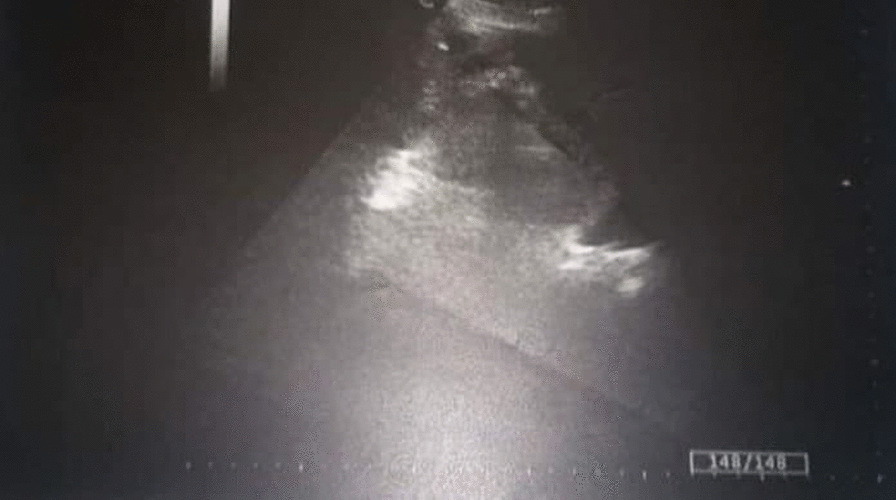
Abdominal Ultrasound showing an accumulation of intestinal loops in the epigastric area

**Fig. 3:**
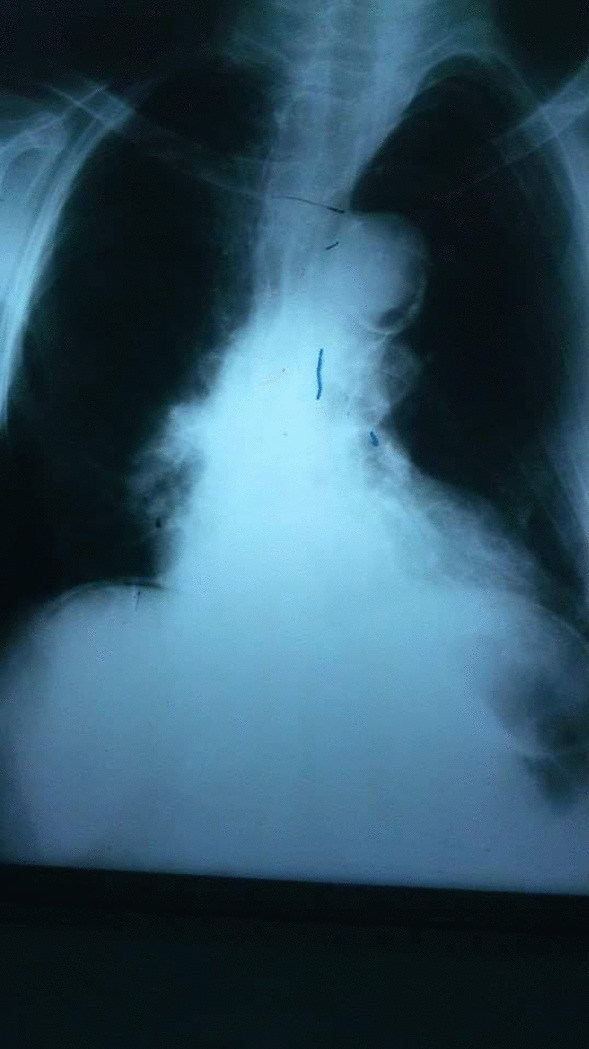
Posterior-Anterior erect chest X-ray showing free gas under the right diaphragm

**Fig. 4:**
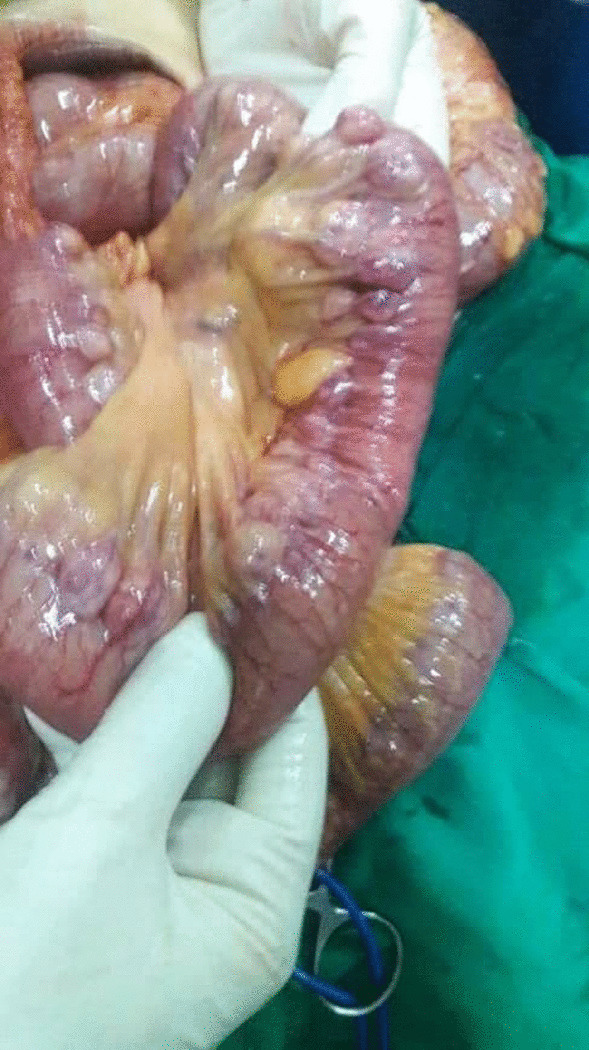
Gross representation of diverticula spreading over the entire small bowels

**Fig. 5:**
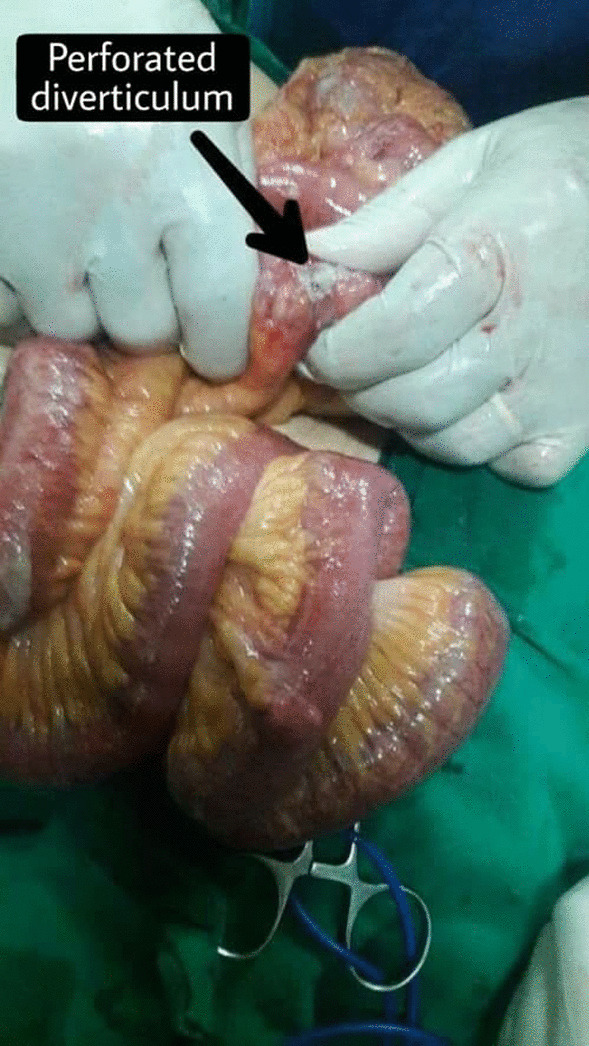
Gross view of the perforated jenjunal diverticulum

**Fig. 6:**
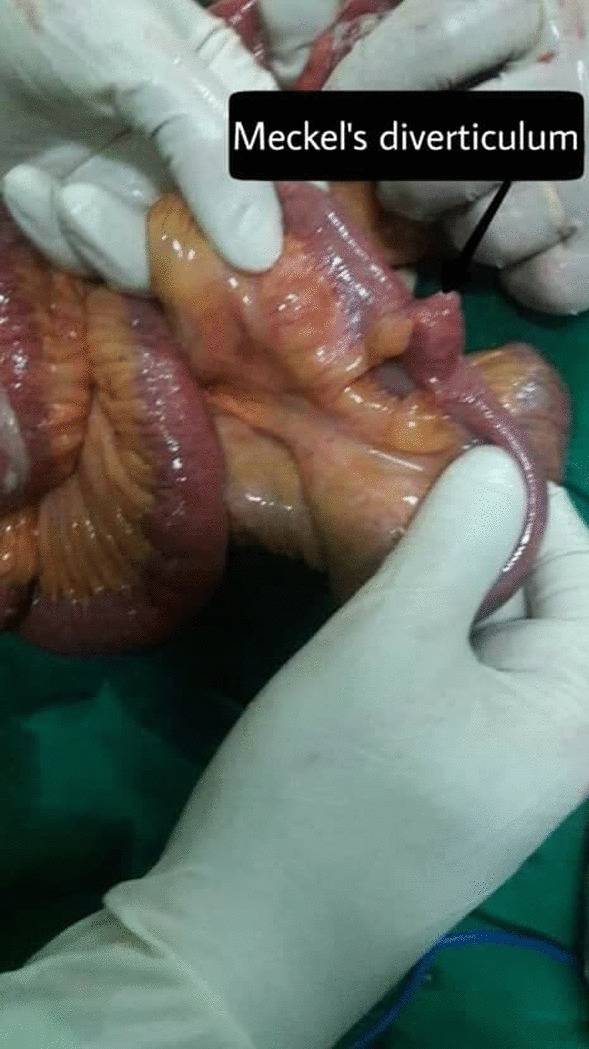
Gross image of Meckel’s diverticulum 70 cm away from the ileocecal valve

**Fig. 7:**
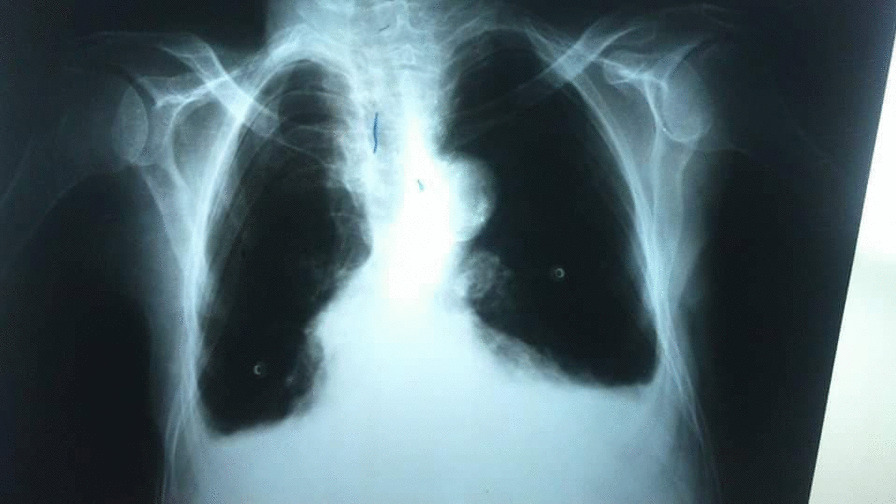
Posterior-Anterior erect chest x-ray showing a bilateral pleural effusion

An 84-year-old female was admitted to the Department of General Surgery complaining of severe, generalized abdominal pain with epigastric intensification. The pain started 24 h before admission and gradually increased. It was accompanied by nausea, but no reported vomiting. A medical history of epigastric pain that worsened one hour postprandial, which after investigations were attributed to gallstones, was observed. Thus, a cholecystectomy after which the pain was not completely relieved. Later on, the patient was diagnosed with a peptic ulcer and put on a proton pump inhibitor. However, the abdominal discomfort persisted. She was also previously diagnosed with atrial fibrillation, mitral valve regurgitation, constipation attributed to her old age, and external hemorrhoids treated conservatively. Medication history consisted of (Aspirin 100 mg, Omeprazole 30 mg, Digoxin 0.25 mg, and Lasix). On admission, the patient was alert with vital signs as follows: (Blood Pressure 120/80 mmHg, Temperature 38.5 °C, Respiratory Rate 20/min, and Heart Rate 98 beats/min). Physical examination revealed a hernia in the epigastric region, marked tenderness in the right hypochondriac area with abdominal guarding. An abdominal Ultrasound was performed reporting a resected gallbladder, a heterogeneous mass in the epigastric area, and a 7 mm epigastric midline hernia (i.e., linea alba hernia) that contained intestinal loops (Figs. 1, 2). A Chest X-ray showed free gas under the right diaphragm (Fig. 3). Laboratory test results showed high levels of red blood cells (7 million cells/mcL), white blood cells (15,800 mcL), C-reactive protein (CRP) (90.2 mg/dL), and low levels of hemoglobin (11.1 g/dL) and albumin (3.1 g/dL). Whereas Creatinine, Bilirubin, Amylase, and Lipase values were within the normal limits (Table 1). An echocardiogram (ECG) reported mitral leaflets vegetations, posterior leaflet prolapse with severe regurgitation, a pulmonary pressure of 65 mmHg, and calcification of the aortic valve. An exploratory laparotomy was performed, via a median incision. Afterward, a cloudy fibrinous exudate was noticed in the abdominal cavity. Exploration of the bowels revealed many small diverticula that spread over the entire small intestines (Fig. 4). One jejunal diverticulum (JD) was perforated (Fig. 5). The omentum was spotted gathered around the perforated JD in the epigastric area, which explained the heterogeneous mass marked on abdominal ultrasound. Diverticulectomy and suturing were applied to the perforated diverticulum. The other intestines’ investigation showed Meckel’s diverticulum 70 cm away from the ileocecal valve (Fig. 6). The large intestines were found spared of any diverticula. IV fluids, Ceftriaxone 1 g q12h, Gentamicin 80 mg q12h, Ranitidine 50 mg q12h, and Acetaminophen 500 mg were administered. Postoperative monitoring confirmed the stability of the patient’s vital signs and general condition improved, and consequently, the patient was discharged 5 days after surgery. The histopathological findings of the 2 × 1.5 × 0.5 cm resected perforated jejunal diverticulum revealed nonspecific acute inflammatory changes with acute inflammatory infiltrate in the surrounding fat tissue. Six days after surgery, a symptom of mild, non-productive cough was reported. On physical exam, chest auscultation findings demonstrated decreased breath sounds at the lung bases, with no rales or wheezing, whereas the vital signs were normal. Oxygen saturation was 96%. A Chest X-ray showed bilateral pleural effusion that was eventually considered a normal post-surgical reaction (Fig. 7).

**Table 1:** Admission laboratory tests results

WBCs	Neutrophils	Lymphocytes	RBCs	Hemoglobin	Total bilirubin
15,800 /mm^3^	90.8%	4.3%	7 million cells/mcL	11.1 g/dL	0.6 mg/dL

Direct bilirubin	Amylase	Total protein	Albumin	Urea	Creatinine	CRP
0.2 mg/dL	35 U/L	5.2 g/dL	3.1 g/dL	25 mg/dL	0.6 mg/dL	90.2 mg/L

*WBC* white blood cells, *RBCs* red blood cells, *CRP* C-reactive protein

## Discussion and conclusion

JD is a disease of elderly people, and over 80% of affected individuals are in the 7th decade of life. The average age of presentation is 62 years, and its incidence is slightly more common in men. According to the literature containing 290 patients, the age of the patients ranged between 45 and 90 years old, with only one case in which the age of the patient was 36. The diverticula incidence in the colon simultaneously with the jejunoileal diverticulum is 20–70%, in the duodenum 10–40%, in the esophagus and stomach 2%. While some patients with complicated JD were asymptomatic [1, 3, 5–12], the rest presented with abdominal pain with features of bowel obstruction such as vomiting and constipation, or with perforation symptoms. With regard to its diagnosis, complicated JD can manifest as a bullion-like appearance on barium radiograph. Although enteroclysis and enterography are the best imaging modalities in the diagnosis of complicated JD, their use in emergency situations is limited. Therefore, radiographs and computed tomography imaging are mostly used. While radiographs can reveal features such as free air under the diaphragm or others [1, 8, 11, 13–18], many were unremarkable [5, 6, 9, 19–23]. However, CT imaging is a more reliable method of diagnosis and was able to show the features of this entity in all cases when it was obtained [1, 4, 7, 9, 10, 13, 15, 17, 22–28]. A diagnostic laparoscopy was also done in some cases [4–6, 8, 12, 14, 15, 19–21, 26, 29]. Rarely, other diagnostic tools such as Ultrasonography were performed [4, 6, 13, 14, 17]. To sum up, a CT scan seems to be the best imaging modality of choice, especially in emergency situations to ensure not to miss any complicated JD cases that can subsequently result in detrimental outcomes. In general, management is not indicated in asymptomatic small bowel diverticulosis. However, the treatment option in symptomatic cases is based on the clinical presentations (antibiotic therapy, restricted diet, and surgical management with open or laparoscopic-assisted resection). In light of our literature review, there were 23 patients who were treated conservatively and 267 patients underwent surgery. Jejunal resection, segmental resection of the jejunum or small bowel resection, with end-to-end or side by side anastomosis, was performed in the majority of cases [1–3, 6–9, 11, 12, 14, 19, 20, 23, 26, 29–31]. In the article by Spasojevic M et al., the authors report 3 groups; group 1 had a review of published cases after 1995 combined with their original data; group 2 had data from the Norwegian Patient Registry, and group 3 had cases reported in the literature before 1995 and were considered as controls. There were no significant differences in the outcomes of conservative or surgical management between the groups. However, there was a difference in the type of surgical procedure, in which surgical resection outcomes were better in group 1 compared to group 3. Whereas the surgical procedure most often performed in group I was small bowel resection (83, 90.1%), followed by suture closure (5, 5.5%), small bowel resection in group III was performed in 31 (67.4%) patients and suture closure in 15 (32.6%) [3]. Lempinen et al. performed jejunal resection with anastomosis in cases 1–6. In addition to appendectomy in case 2, whereas the patient in case 8 underwent excision of the fistula and end-to-end anastomosis. However, there was no resection in case 7, only adhesiolysis and decompression [4]. There were some cases where the resection was not required [16, 18], therefore, the surgical procedure included repairing the perforations. A laparotomy diverticulectomy for a perforated diverticulum with a single-layer duodenal closure was the treatment of choice in one case [10]. Additional large non-inflamed widely spaced diverticula were discovered in another case and were left not excised [7]. The conservative treatment may be recommended in cases with surgery contraindications [17, 22, 27] or as initial management of an acute attack of diverticulitis [6]. It was mainly based on intravenous/oral antibiotics and anti-inflammatory medications, including Prednisolone, Ciprofloxacin, Piperacillin-Tazobactam, Metronidazole, Trimethoprim, Sulfamethoxazole, and Levofloxacin. By follow-up, most cases were discharged alive 1-week post-operation. Mortality was significantly higher in Group III (23.4%) compared to Group I (5.7%) [3]. In addition, two patients died as reported in case 5 on 17 days post-operation [4], and in case 3 of an 85-year-old patient with esophagus adenocarcinoma, even though it was asymptomatic for 1-year post-operation before presenting with unrelated transient small intestine obstruction [27]. Long-term doxycycline was prescribed in case 2 [27]. Moreover, patients were discharged in 2–48 days of operation, the patient was discharged on day 48 in case 6 [4] while the hospitalization period was only 2 days in case 4 [27]. A few months of follow-up, CT revealed no extraperitoneal air or mesenteric infiltration [17]. Overall, no serious complications or recurrence was observed in the majority of cases except an episode of aspiration pneumonia [16], hospital-acquired pneumonia [11], swelling of the hand joints, polyarthralgia, fatigue 3 days post-operation [14], and wound infection at suture site [3, 8]. With that in mind, small bowel diverticula can have several complications such as bleeding, obstruction, and diverticulitis that can lead to perforation [32–34]. In the current paper, we provide a case of perforated jejunal diverticulitis. Perforation is one of the rarest complications (2.1–7% of diverticulitis cases), but carries a great risk with high mortality rates [32, 34]^.^ The clinical presentation in the cases of perforation is mostly acute with symptoms and signs similar to peritonitis such as fever and severe abdominal tenderness. Complications of diverticula can be managed conservatively or surgically, mainly in cases of perforation [32–34]. The extent of resection can cause further consequences, especially in cases with extensive diseases involving large parts of the bowel. Therefore, clinical evaluation is required to avoid short bowel syndrome [25] (Table 2). Furthermore, the role of interventional radiology in the management of complicated small bowel diverticula has been growing, especially in managing cases of bleeding [35]. Therefore, we recommend consulting interventional radiology when encountering cases of complicated diverticula. In conclusion, resection of the small bowel diverticulum and repair of the perforations should be considered for the management of these cases, resulting in a high rate of survival and good outcomes. Antibiotics could be prescribed to avoid complications. The conservative treatment may be suggested for patients with surgery contraindications.

**Table 2:** .

Reference N	Patient age (y)/ sex	Chief complaint	Diagnostic tests	Findings	Surgical management (Rationale)	Conservative management (Rationale)
1	C1: 36/F C2: 75/F	C 1: Abd pain, N&V C 2: Abd pain, N&V and fever	AXR, Abd CT	C 1: AXR: air under the diaphragm, Abd CT: free air, fluid collection, and edema in the mesentery C 2: AXR: N, Abd CT: no free air, no fluid collection, edema in the small bowel loops	C1: Laparotomy: segmentary small bowel resection, side-by-side anastomosis C 2: Laparotomy: segmentary small bowel resection, side-by-side anastomosis	
2	90/ F	Abd pain	Abd CT	Perforated jejunal diverticulum with abscess formation	Laparotomy	
3	Group I (106 pts):the mean age was 72.2 ± 13.1 y/F,M Group II (113pts): the mean age was 67.6 ± 16.4 y/F,M Group III (47 pts): the mean age was 65.4 ± 14.4 y/F,M	Group I: moderate fever (46.9%), no fever (26.5%), high fever in 26.5%	AXR, Abd CT, and exp laparotomy		Group I: 92 pts underwent surgery: small bowel resection (83, 90.1%), followed by suture closure (5, 5.5%). Two patients (2.2%) underwent complex procedures that included multiple resections and 2 (2.2%) underwent surgical exploration with drainage Group II: laparotomy: small bowel resection in 93 (82.3%) patients and enterorrhaphy in 17 (15%) Group II: 46 pts underwent surgery: Small bowel resection was performed in 31 (67.4%) pts and suture closure in 15 (32.6%)	Group I: 14 pts were treated conservatively Group II: only one pt was treated conservatively
4	Range 59–83 /F,M	Abd pain	Abd CT, Exp laparotomy	pt1: extensive jejunal diverticulosis, adjacent mesenteric abscess, pt2: single jejunal diverticula with an adjacent mesenteric abscess, pt3: free air in the abdomen, faecal peritonitis and multiple jejunal diverticula, pt 4: Occlusion, solid tumour, pt5: faecal peritonitis and diverticula perforation, pt6: multiple jejunal diverticula and an abscess, pt7: occlusion, pt8: multiple jejunal diverticulosis and a jejuno-colic fistula	Laparotomy: Resection of the involved jejunal segment with primary anastomosis was performed in 6 of the 7 patients with acute symptoms. In patient 7 laparotomy with decompression only was performed because of adhesiolysis. Pt 8: Nefrectomy. Excision of fistula and end to end anastomosis	
5	Middle aged/ M	Abd pain	AXR, Abd CT	AXR: N Abd CT: a large calcified mass within the lumen of the small bowel, with evidence of mesenteric twist or volvulus	Laparotomy: segmentary small bowel resection, side-by-side anastomosis	
6	C1: 74 /M C2: 65 /F	C1: Abd pain and vomiting C2: Abd pain, vomiting, and anorexia	C1: AXR C2: AXR and laparoscopy	C1: AXR: N C2: AXR: dilated small bowel loops in upper abdomen, Diagnostic laparoscopy: multiple interloop adhesions	C2: Laparotomy: Laparoscopic adhesiolysis with resection of involved segment and jejuno-jejunal anastomosis	C1: Conservatively
7	59 /F	Abd pain	Abd CT	Jejunal loop with a large diverticulum on the mesenteric side with diverticulitis and perforation	Laparotomy: segmentary small bowel resection, side-by-side anastomosis 4 other large non-inflamed diverticula are not excised, as this would have required multiple further small bowel resections and anastomoses with associated increased morbidity	
8	50 /M	Abd pain and nausea	AXR, Exp laparotomy	AXR: multiple air fluid levels At surgery: multiple jejunal diverticula with a perforation in one of the diverticulum	Laparotomy: segmentary small bowel resection, side-by-side anastomosis	
9	82 /M	Abd pain and nausea	Abd CT	A hollow viscus perforation with intra-abd free air and intra-pelvic free fluid	Laparotomy: segmentary small bowel resection, side-by-side anastomosis	
10	80/F	Abd pain and vomiting	Abd CT	fluid and gas surrounding the second and third portions of the duodenum, thickening of the duodenal wall, retroperitoneal fat stranding and perihepatic free fluid	Laparotomy: diverticulectomy with single-layer closure was performed	
11	74 /F	Abd pain, N&V	CXR, AXR	free gas under the right hemidiaphragm and nonspecific gaseous distension of the small bowel	Laparotomy: Resection of the involved jejunal segment and a primary jejunal anastomosis were performed	
12	63/M	Abd pain	AXR, Abd CT	AXR: non-specific gaseous distension of the large and small bowel Abd CT: an area of apparent communication between right-sided loops of small bowel with visualised extraluminal gas, a calcific focus noted central to the involved segment	Exploratory laparotomy: segmentary small bowel resection, side-by-side anastomosis (On presumption of perforation)	
13	56 /M	Abd pain	AXR, Abd CT	AXR: air under the diaphragm Abd CT: multiple diverticula in the small intestine and air under the diaphragm suggesting perforation	Laparotomy (Radiological investigations suggested perforation)	
14	70 /M	Abd pain	AXR, Exp laparotomy	AXR: air-fluid levels with several dilated loops in the small bowel, but no free peritoneal air Exploratory laparotomy: multiple diverticulosis with a large inflammatory reaction covering a perforated diverticulum	Exploratory Laparotomy (bowel infarction, perforation, necrosis, ischemia and uncontrolled severe abdominal pain)	
15	74 /F	Abd pain, N&V	AXR, Abd CT	AXR two gas fluid lesions in the small intestine Abdominal CT: multiple diverticula on the mesenteric wall of the small intestine and dilated intestinal loops proximal to the diverticula, but no free air or fluid	Exploratory laparotomy (acute symptoms)	
16	82 /F	Abd pain and vomiting	AXR, Abd CT	AXR: multiple dilated loops of small bowel Abd CT: multiple small bowel diverticula were identified with surrounding pockets of free air adjacent to the jejunal diverticula suggestive of a small bowel perforation	Laparotomy (Abd CT suggested perforation)	
17	80/F	Abd pain	AXR, US, Abd CT	AXR: dilated small bowel loops US: two hypoechoic irregular formations Abd CT: thickening of the jejunal wall, air bubbles and localized perforation		Conservatively (antibiotic therapy)
18	50/M	Abd pain and nausea	AXR and exp laparotomy	AXR: no free gas under diaphragm and multiple air fluid levels	Exploratory laparotomy (signs of peritonitis, AXR)	
19	74/M	Abd pain, constipation, anorexia and fever	CXR, AXR, Exp laparotomy	CXR: N AXR: prominent but non-dilated small bowel loops	Emergency laparotomy (acute symptoms)	
20	76/F	Abd pain and confusion	AXR, Abd CT, Exp laparotomy	AXR: N Abd CT: a ring enhancing collection, air-fluid level, extensive adjacent mesenteric inflammation, thickened and edematous mid-jejunum loop, intraperitoneal free air, perforated jejunal diverticulitis, abscess, no bowel obstruction/ascites	Laparotomy (Abd CT findings suggested the perforation)	
21	74/M	Abd pain and distention, fever	CXR, AXR, Exp laparotomy	CXR, AXR: N Abd CT: extraluminal air, abscess adherent to jejunum	Laparotomy: (Partial enterectomy of 45 cm jejunum including the diverticula and side-to-side anastomosis)	
22	63/F	Non-specific abd pain	AXR, CXR, Abd CT	AXR, CXR: N Abd CT: jejunal diverticulitis surrounded with inflammatory infiltrate and small jejunal diverticula		Conservatively (due to the patient’s comorbidities)
23	79/F	Abd pain, fever, chills	CXR, Abd CT	CXR: N Abd CT: extraluminal air	Laparotomy: (resection of involved jejunum and end-to-end anastomosis)	
24	85/M	Abd pain, hypotension, peritonitis signs	Exp laparotomy	Exp laparotomy: peritoneal contamination, colonic pseudodiverticula, perforated jejunal pseudodiverticulum	Laparotomy (resection of involved jejunum and end-to-end anastomosis)	
26	90/M	Abd pain, N&V and diarrhea	Abdominal CT, Exp laparotomy	Abd CT: inflammation, pneumoperitoneum	Laparotomy: small bowel resection with hand-sewn anastomosis	
27	pt1: 87/M pt2: 86/F pt3: 78/F pt4: 76/M	pt1: Abd pain and fever pt2: Abd pain pt3: Abd pain and diarrhea pt4: Abd pain and constipation	AXR: pt1, pt4 Abd CT: pt1-4 Endoscopy: pt2	AXR: pt1 N pt4: prominent small intestine loops, air-fluid levels Endoscopy: pt2: 2 large diverticula Abd CT: pt1: multiple diverticula, circumferential thickening and gas, perforated diverticulitis pt2: inflammatory mass pt3: diverticulitis, no perforation pt4: inflammation, localized luminal air, numerous diverticula		Conservatively Pt2: (patient's symptoms resolved relatively quickly and because the patient had no sign of free perforation on imaging) Pt3: patient's age and other comorbidities)
28	pt1: 79/F pt2: 87/F pt3: 77/M	pt1: Abd pain pt2: Abd pain pt3: Abd pain	AXR: pt1, Abd CT: pt1-4	AXR: pt1: N Abd CT: pt1: colonic diverticulosis and scattered jejunal and ileal diverticula, ​jejunal diverticulitis pt2: scattered jejunum and ileum diverticulum, two extraluminal foci of air, perforated diverticulitis pt3: multiple colonic diverticula, small obstructed diverticulitis	pt1: Surgery pt2: Conservatively and surgery Pt3: Not reported	
29	74/M	Abd pain, nausea and flatulence	Exp laparotomy	Exp laparotomy: multiple jejunal diverticula, ruptured diverticula, peritonitis	Laparotomy: jejunal segment resection, and anastomosis (suspicion of perforation)	
30	83/F	Abd pain	CXR, Abd CT	CXR: no free subdiaphragmatic gas Abd CT: multiple diverticula and free gas	Laparotomy: jejunal segment resection, and anastomosis (perforation)	
31	79/M	Abd pain	Abd CT	Abd CT: distal jejunal loop thickening and infiltration, free air	Laparotomy: jejunal segment resection, and anastomosis (perforation)	
32	82/M	Abd pain and nausea	Abd CT	Abd CT: revealed fluid collection, air bubbles around the duodenum		Conservatively (patient’s age, absence of peritonitis, and stable clinical condition)

## Data Availability

Not applicable. All data (of the patient) generated during this study are included in this published article and its additional information files.

## Abbreviations

CT: Computed tomography
MRI: Magnetic resonance imaging
ERCP: Endoscopic retrograde cholangiopancreatography
ECG: Echocardiogram
JD: Jejunal diverticulum
CRP: C-reactive protein
